# Can Replication Save Noisy Microarray Data?

**DOI:** 10.1002/cfg.196

**Published:** 2002-08

**Authors:** Lorenz Wernisch

**Affiliations:** School of Crystallography Birkbeck College London WC1E 7HX UK

## Abstract

Microarray experiments are multi-step processes. At each step—the growth of cultures, extraction of mRNA, reverse transcription, labelling, hybridization, scanning, and image analysis—variation and error cannot be completely avoided. Estimating the amount of such noise and variation is essential, not only to test for differential expression but also to suggest at which level replication is most effective.

Replication and averaging are the key to the estimation as well as the reduction of variability. Here I discuss the use of ANOVA mixed models and of analysis of variance components as a rigorous way to calculate the number of replicates necessary to detect a given target fold-change in expression levels. Procedures are available in the package YASMA (http://www.cryst.bbk.ac.uk/wernisch/yasma.html) for the statistical data analysis system R (http://www.R-project.org).


**Comparative and Functional Genomics**
*Comp Funct Genom* 2002; **3**: 372–374.
Published online in Wiley InterScience (www.interscience.wiley.com). **DOI:** 10.1002/cfg.196

**Conference Review**

### Can replication save noisy microarray data?

Lorenz Wernisch*
*School of Crystallography, Birkbeck College, London WC1E 7HX, UK*

**Correspondence to:*
*Lorenz Wernisch, School of Crystallography, Birkbeck College, London WC1E 7HX, UK.*
*E-mail:*
*l.wernisch@cryst.bbk.ac.uk*

Received: 31 May 2002
Accepted: 12 June 2002

### Abstract

**Microarray experiments are multi-step processes. At each step — the growth of cultures, extraction of mRNA, reverse transcription, labelling, hybridization, scanning, and image analysis — variation and error cannot be completely avoided. Estimating the amount of such noise and variation is essential, not only to test for differential expression but also to suggest at which level replication is most effective.**

**Replication and averaging are the key to the estimation as well as the reduction of variability. Here I discuss the use of ANOVA mixed models and of analysis of variance components as a rigorous way to calculate the number of replicates necessary to detect a given target fold-change in expression levels. Procedures are available in the package YASMA (http://www.cryst.bbk.ac.uk/wernisch/yasma.html) for the statistical data analysis system R (http://www.R-project.org). Copyright © 2002 John Wiley & Sons, Ltd.**

### The power of averaging

In the following, I assume that the results of a replicated microarray experiment are $\log_2$ ratios of fluorescent intensities in the two channels of a two-dye experiment comparing an experimental condition with a control condition. Any necessary background correction and normalization has been applied and each gene is associated with a series of $\log_2$ ratio values from replicated experiments. The statistical assumption is that $\log_2$ ratio values are normally distributed around their true mean. Genes with no differential expression have a true mean of 0. Any deviation of a mean $\log_2$ ratio from this base line indicates over- or underexpression.

Averaging is the key to the estimation of the true mean. Figure 1 demonstrates the fact — well-known from elementary statistics — that the average of a growing number of replicates approaches the true mean with decreasing variability. It shows 50 simulations from a normally distributed random variable with mean $\mu = 0.5$ and variance $\sigma^2 = 1$. The sample means of increasingly larger samples are indicated by open circles connected by a line. Notice that even though the variability is large (if these were $\log_2$ ratios of a real gene its fold-change would range from eight-fold under- to eight-fold overexpression), the true mean can be reliably estimated, provided enough replicates are considered. The reason for this improved accuracy with replication is that the variance $\sigma_m^2$ of the sample mean is inversely proportional to the number $n$ of replicates, $\sigma_m^2 = \sigma^2/n$.

### How many arrays do I need?

The exact number of replicates necessary to detect overexpression of a gene reliably depends on several parameters. First, the underlying variance $\sigma^2$ must be obtained (in the next section I discuss variance estimates from microarray data). Next, a significance level $\alpha$ needs to be specified for the probability of a type I error, i.e. the probability that a neutral gene with zero mean is falsely called as overexpressed. A gene with sample mean $m$ will be *called*, if $1 - \Phi(m/\sigma_m) \leq \alpha$ (where $\Phi$ is the standard normal cumulative distribution function). Finally, a minimum $\log_2$ ratio value $t$ (target change) for overexpression, as well as a level $\beta$

Copyright © 2002 John Wiley & Sons, Ltd.

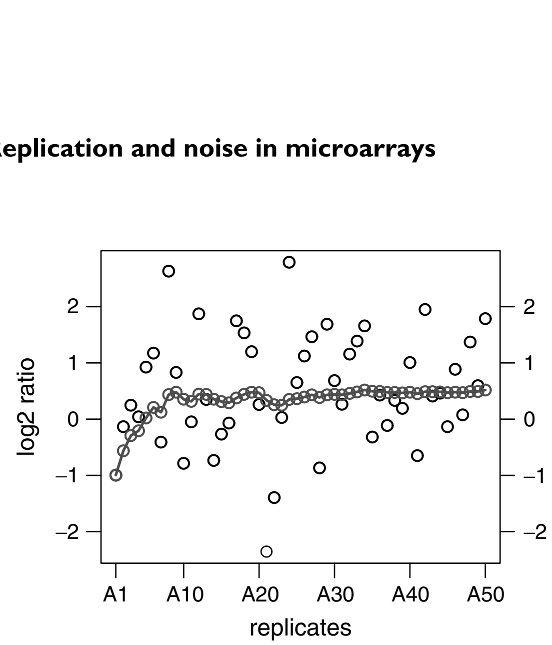

**Figure 1.** Sample means of increasingly larger samples of a normally distributed random variable with mean 0.5 and variance 1

for the type II error of not calling an overexpressed gene, must be provided.

The probability $p$ that a gene with true mean 0 has a sample mean of at least $t_0$ is:

$$p = 1 - \Phi\left(\frac{t_0}{\sigma_m}\right)$$

where $\sigma_m$ is the variance of the sample mean and $\Phi$ is the standard normal distribution. Similarly, the probability $q$ that a gene with true mean $t$ has a sample mean smaller than $t_0$ is:

$$q = 1 - \Phi\left(\frac{t - t_0}{\sigma_m}\right)$$

From the requirement that $p \leq \alpha$ and $q \leq \beta$, after a few transformations we obtain an upper limit for the acceptable variance of the sample mean:

$$\sigma_m \leq \frac{t}{\Phi^{-1}(1-\alpha) + \Phi^{-1}(1-\beta)} \tag{1}$$

With $\sigma$ given, a minimum number of replications $n$ is now easily derived from $\sigma_m = \sigma/\sqrt{n}$. (If there are only a few degrees of freedom for the estimation of $\sigma$, then $\Phi$ needs to be replaced by the $t$ distribution. If $n$ is part of the calculation of the degrees of freedom, the above equation needs to be solved in an iterated fashion.)

In the above example, $\sigma = 1$. If the target $t$ is a $\log_2$ ratio value of 0.5 (1.41 fold-change), which should be detectable at a significance level of 0.05, then $n \geq 10.82$, i.e. 11 replicates are needed if we accept that half of the overexpressed genes might go unnoticed ($\beta = 0.5$, although this is a very conservative estimate). If we want to detect at least 75% of overexpressed genes ($\beta = 0.25$), the number of necessary replicates rises to 22.

### Hierarchical replication

Microarray experiments are done in stages, and at each stage replication can be used for averaging and reduction of variation. Examples are the growing of several cultures of the same mutant, multiple mRNA extractions, multiple spots on one array, or even multiple image analyses. Considering that replications on each level come at different costs, estimating their relative contributions to overall variation can help designing cost-efficient experiments.

To be more specific, let us assume that replication has been conducted in the following way. $n_C$ cultures are grown separately, each one hybridized on $n_A$ arrays, and each array scanned and analysed as image $n_I$ times. This results in a total of $n_C n_A n_I$ array data sets replicated in a hierarchical fashion (hybridizations nested in cultures, scannings nested in hybridizations). One difficulty with the analysis of such experiments is that replicates are no longer independent of each other, e.g. replicates from the same culture will show some correlation in their noise, due to the common underlying variation in the culture.

Assuming linearity of effects and a normal distribution of noise, an *analysis of variance components* allows us to calculate the variance contributions at different levels in such hierarchical designs (see Oehlert [1]) for an accessible introduction to this topic). Analysis of variance components is similar to a standard ANOVA analysis but recognizes that some effects are random. For example, if the same culture was used in all future microarray experiments, the culture effect would be a *fixed* factor in an ANOVA analysis. The likelier scenario is that new cultures are grown every time, and the culture effect enters the analysis as a *random* factor. Usually, we are interested in noise inherent in the process of growing new cultures or hybridizing to new arrays. Consequently, such factors are best analysed as random factors.

In the hierarchical experimental setting described above, the overall variance of cultures $\sigma_C^2$ and

Copyright © 2002 John Wiley & Sons, Ltd. 

the overall variance of arrays $\sigma_A^2$ are usually very small and can be ignored once raw intensity data have been normalized. What remains are the variances related to genes, such as the gene-culture variance $\sigma_{GC}^2$ the gene-array variance $\sigma_{GA}^2$ and the residual variance $\sigma^2$ stemming from multiple image analyses. These variance components all contribute to the variance $\sigma_m^2$ of the mean of $\log_2$ ratios for a particular gene:

$$\sigma_m^2 = \frac{\sigma_{GC}^2}{n_C} + \frac{\sigma_{GA}^2}{n_C n_A} + \frac{\sigma^2}{n_C n_A n_I} \qquad (2)$$

If such variance components have been derived from preliminary studies, then this equation combined with equation 1 allows the calculation of the number of cultures, of arrays per culture, and of image analyses per array, necessary to achieve a desired resolution in differential gene expression. Simply increasing the number $n_C$ of cultures is the best way to decrease variability. If different costs are involved in producing cultures and arrays or image analyses, then some balance between numbers of cultures, arrays and image analyses might be better. The best combination of such numbers can be obtained by optimizing $\sigma_m^2$ under the additional constraints on costs.

### Acknowledgements

I acknowledge BμG@S (the Bacterial Microarray Group at St George's) and The Wellcome Trust for funding their work.

### Reference

1. Oehlert GW. 2000. *A First Course in Design and Analysis of Experiments*. W. H. Freeman: New York.

Copyright © 2002 John Wiley & Sons, Ltd. 

